# Quantitative Electroencephalography Analysis (qEEG) of Neuro-Electro-Adaptive Therapy 12™ [NEAT12] Up-Regulates Cortical Potentials in an Alcoholic during Protracted Abstinence: Putative Anti-Craving Implications

**DOI:** 10.4172/2155-6105.1000171

**Published:** 2014

**Authors:** Roger L Waite, Marlene Oscar-Berman, Eric RBraverman, Debmalya Barh, Kenneth Blum

**Affiliations:** 1 Department of Nutrigenomic Research, Bio-Clarity, LLC, San Diego, California USA; 2 Department of Psychiatry and Neurology, Boston University School of Medicine and Veterans Administration System, Boston, Massachusetts, USA; 3 Department of Psychiatry, University of Florida, College of Medicine and McKnight Brain Institute, Gainesville, Florida, USA; 4 Center for Genomics and Applied Gene Therapy, Institute of Integrative Omics and Applied Biotechnology (IIOAB),Nonakuri, Purba Medinipur, West Bengal, India; 5 Department of Addiction Research & Therapy, Malibu Beach Recovery Center, Malibu Beach, California, USA; 6 Dominion Diagnostics, LLC, North Kingstown, Rhode Island, USA; 7 Path Foundation NY, New York, New York, USA

## Abstract

**Introduction:**

Cranial electrotherapy stimulation (CES) is a noninvasive therapy that has been used for decades in the United States to treat anxiety, depression, and insomnia in the general population. The effectiveness of CES has been questioned by many and its use is considered controversial. In this study we are presenting data on one alcoholic patient using a newly engineered device we call Neuro-Electro-Adaptive Therapy 12™ [NEAT12]. This hybrid device utilizes TENS current characteristics yielding CES effects. This device has been found to primarily target the excitation of the Cingulate Gyrus region of the brain.

**Case presentation:**

This is a 42 year old male who has been abstinent from alcohol for approximately two months. The data presented herein represents the pre to post qEEG differences of an alcoholic in protracted abstinence. This subject was evaluated both before and after using the NEAT-12 device. The pre to post comparisons suggest that the cortical potentials especially at the Cingulate Gyrus are up regulated after using the device. The absolute power changes obtained shows a decrease of more than 2 SD as noted in the delta wave spectrum. Also noted is an overall cortical increase in the alpha spectrum. The resting alert state of a neuro typical population is most prominently marked by a regulation of 7.5-11 Hz alpha throughout the cortex. The decreased in delta and theta suggests an up regulation of the prefrontal cortex and the anterior Cingulate Gyrus a site involved in substance use disorder (SUD).

**Conclusion:**

A presence of dominant slow waves through the prefrontal cortex and the anterior Cingulate Gyrus is often associated with OCD, anxiety, impulsivity and cravings in addicted populations. It is conceivable that our initial finding of altered electrical activity of the brain using qEEG analysis suggests the NEAT-12 may induce a “normalization” of aberrant electrical activity of the cortical region of the brain known to occur during protracted abstinence of alcoholics. It may have utility as a putative anti-craving CES device and therefore warrants intensive investigation.

## Introduction

The Cranial Electrical Stimulation (CES) technique appeared at the beginning of the 1960s and is aimed to act at the level of the central nervous system [1]. The current, composed of high frequency pulses interrupted with a repetitive low frequency, was delivered through three electrodes (a negative electrode placed between the eyebrows while two positive electrodes are located in the retro-mastoid region). We have recently introduced a new CES Device, the NEAT-12, in which shortcomings encountered with previous electrical stimulation techniques are avoided due to changes in the characteristics of the delivered current. The main property of CES is to potentiate some drug effects, especially opiates and neuroleptics, during anesthetic clinical procedures [2]. This potentiation effect permits a drastic reduction of pharmacological anesthetic agent and results in reduced post-operative complications. Despite numerous clinical and animal studies performed with this technique for several decades, CES mechanisms are not completely elucidated. Animal studies demonstrated that stimulationwith CES releases 5-hydroxy-indol-acetic acid and enkephalins [2]. These results obtained without any undesirable outcome are encouraging signs. Continued investigation of this electrotherapeutic technique is warranted.

## Brief History

CES received U.S. Food and Drug Administration (FDA) approval for the treatment of insomnia, depression, and anxiety in 1979 [3]. CES is the United States FDA term for the transcranial application of small amounts of electricity, usually less than 300-600 mA with a frequency of 100 Hz or lower. CES was imported into the United States from Europe and was originally introduced as electro sleep, possibly because it increases delta waves. CES and electronic medicine did not receive a particularly warm reception in American medicine until clinicians began to utilize the transcutaneous electrical nerve stimulation (TENS) devices for pain. Electrotherapies have been used in psychiatry in the form of electroconvulsive therapy (ECT), which is still utilized in organic brain diseases such as Parkinson’s and organic-based depressions [4].

Scrutiny of Table 1 indicates that abundant research has established that an electroencephalogram (EEG) recorded from a drug abuser has a predictable distributionof electrical power (measured in micro-volts squared), just as does the electrocardiogram (EKG). The predictable electrical signals recorded by the EEG, distinctive for each brain region, are regulated by the homeostasis of a complex neuroanatomical brain system that utilizes all known neurotransmitters. Just as the EKG can be used to assess heart dysfunctions, the EEG can assess a wide variety of brain dysfunctions related to developmental, neurological and psychiatric disorders, whether caused by structural or functional abnormalities.

Electrophysiological imbalances secondary to drug abuse have been well characterized (Table 1). These EEG abnormalities are similar in characteristic to the electrophysiological imbalances of various psychiatric diseases such as generalized anxiety, ADHD, depression, OCD and other impulsive disorders.

Abnormal behaviors involving dopaminergic gene polymorphisms often reflect an *insufficiency of usual feelings of satisfaction*, or Reward Deficiency Syndrome (RDS). RDS results from a dysfunction in the “brain reward cascade,” a complex interaction among neurotransmitters (primarily dopaminergic and opioidergic). Individuals with a family history of alcoholism or other addictions may be born with a deficiency in the ability to produce or use these neurotransmitters.

A number of earlier studies have examined the effect of CES on pain, headaches, fibromyalgia, smoking cessation, closed head injuries, and opiate withdrawal [22-24]. There have been a number of studies including some randomized double-blind, controlled experiments [25-31] on generalized anxiety and related symptoms using CES.

## Substance use Disorder and CES

The use of CES as a potential modality for drug abuse was reviewed many years ago by Blum’s laboratory [32]. At that time we showed that in high risk drug abusers the P300 amplitude increased significantly (n=14, P<0.03) when the pre and post qEEG’s were compared. The CES device at a frequency of 100 pulses/sec.1.0 mA, 20% duty cycle, in a square wave form, was worn on the forehead and wrist for 40 minutes. Moreover, fifteen drug abusing individuals were evaluated in an eyes closed and eyes open computerized power spectral analysis for delta, theta, alpha, beta activity before and after 40 minutes of CES. Significant electrophysiological changes occurred in all of the subjects with abnormal baselines. When the pre and post-CES brain electrical activity mapping were compared, significant increases in delta, alpha and beta activity occurred, with a significant decrease in theta activity. We suggested that CES is a therapy that may beneficially alter the abnormal electrophysiology associated with drug abuse and it may normalize patterns of electrotherapy. Others have also studied the effects of CES and drug abuse [33-36]. Specifically, in a double blind placebo controlled investigation [35] it was shown that CES, comprising of the combination of a constant current with a pulse current of square impulses of 70-80 Hz, is an effective method to correct affective disturbances (anxiety, depression) in alcoholic patients. REF Krupitsky et al. [35] in Russia found that the medical effects of CES are accompanied by changes in the metabolism of GABA and monoamines, but not of beta-endorphin. These changes were accompanied by a decrease in the latency of alpha-rhythm appearance eyes closed qEEG.

While there have been a number of studies some of which are negative on the use of CES for the treatment of anxiety, insomnia and depression less is known about the use of CES in substance use disorder [37-39]. In fact there have been no studies related to Substance Use Disorder (SUD) in humans that we can find by doing a PubMed search (7-21-10) after 2000.

The objective of this study was to preliminarily explore whether a newly developed CES device as an effective therapy for patients with DSM-IV diagnosed SUD. This novel waveform is characterized by pulse width and current that is constant reacting independently on the resistant load. Our primary hypothesis was that Neuro-Electro-Adaptive Therapy 12™ [NEAT-12] would reverse dysynchronization of the orbital-prefrontal cortical electro-activity classically observed in protracted abstinence alcoholics. This device utilizes TENS current characteristics yielding CES effects. This is the first report describing not only the device but its putative benefits especially at the Cingulate Gyrus of the brain.

## Method

### Subject

The male was 42 years old with a two month period of sobriety. The subject was recruited from G & G Holistic Addition Center alumni group. He was diagnosed using DSM –IV criteria for SUD with emphasis on alcoholism. He was currently employed but complained about anxiety and a continuation of craving, “white knuckle” sobriety.

### Study design

The study was approved by the PATH Foundation NY Institutional Review Board as part of an approved research project related to genetic testing and novel approaches to SUD treatment (RB NIH registration is # (IRB00002334). The patient signed a consent statement prior to the experiment. The patient was not taking any psychoactive medication at the time of the evaluation. He was drug free as assessed by urine testing. A qEEG analysis obtained one-day prior to NEAT-12 treatments was considered as the pretest. At the exact same time only one day later the patient utilized the NEAT 12 for a one-hour period. Immediately after this treatment the subject was re-evaluated by qEEG analysis.

**Figure 1:**
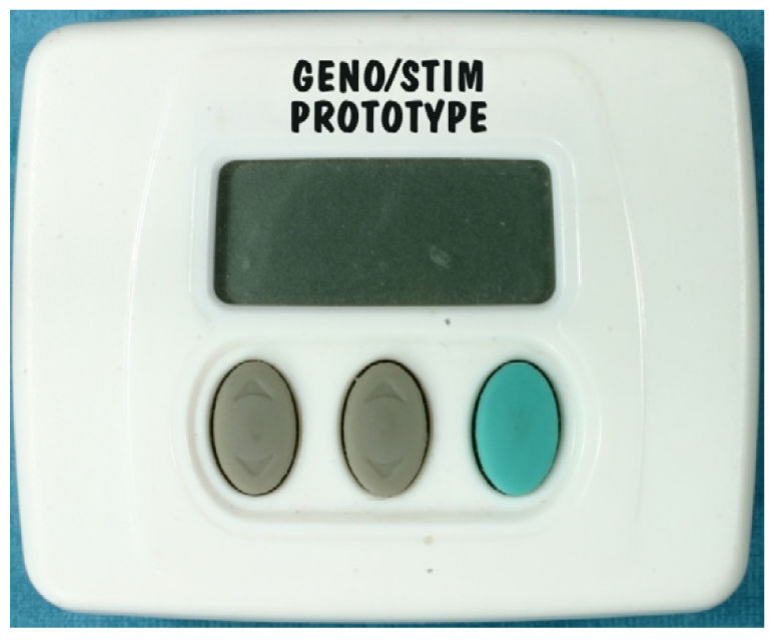
Description of NEAT Device and treatment protocol

The NEAT 12 has twelve independent programs associated to patient’s condition whether it is acute, sub-acute or chronic. The program used in this case study was for post chronic conditions. The current characteristics were 25 uA, at a frequency of 0.44 Hz, polarity change 2.25 seconds every 17 pulses with a modulated pulse. The electric parameters for the NEAT-12 device are shown in Table 1 and Figure 1.

The memory of the NEAT-12 device stores data for 12 different therapeutic waveforms and pulse sequences, each of which is described in Table 2.

In a current search of the existing devices approved for market by the FDA for Cranial Electrical Stimulation no device was found to have any similar waveform characteristics. Many claimed to have waveform harmonics which are trade secrets but from our research the duration, polarity and modulation of our waveform has very little resemblance of any other device on the market today.

### qEEG procedure

Nineteen electrodes using an electro-cap consistent with the International 10/20 systems were placed. Routine EEG was recorded on a Cadwell Easy II using a linked ear montage and with electrodes digitally referenced to the Cz electrode allowing for retrospective montage analysis of all data. Using data gathered under technical conditions as listed above, 59.99 seconds of EEG were selected and subjected to quantitative analysis of absolute power, relative power, power asymmetry and coherence. These measurements are logarithmically transformed and referenced to age-adjusted population norms.

## Results

**Figure 2:**
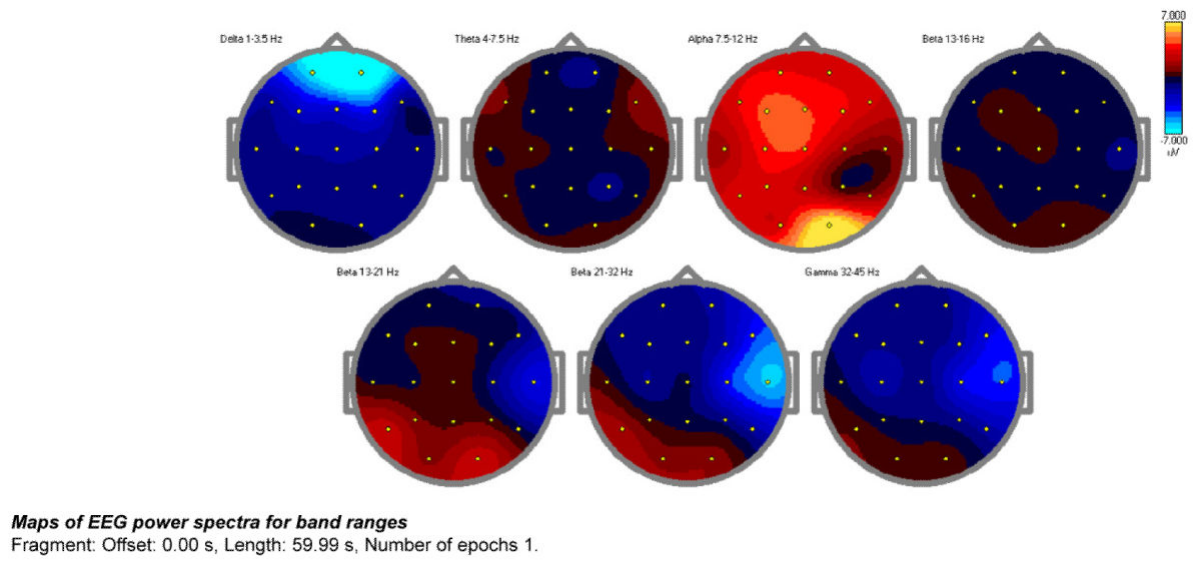
QEEG analysis comparing pre-to post NEAT-12 in alcoholic patient.

Frequency band magnitude (uV) topographies of the EC condition are presented below (Figure 2).

The absolute power changes represented in the above images shows a decrease of more than 2 SD as noted in the delta wave spectrum. Also noted is an overall cortical increase in the alpha spectrum. The resting alert state of a neuro typical population is most prominently marked by a regulation of 7.5-11 Hz alpha throughout the cortex. The decrease in delta and theta suggests an up regulation of the idling frequencies of the prefrontal cortex and the anterior Cingulate Gyrus. A presence of dominant slow waves through the prefrontal cortex and the anterior Cingulate Gyrus is often associated with OCD, Anxiety, and impulsivity.

**Figure 3:**
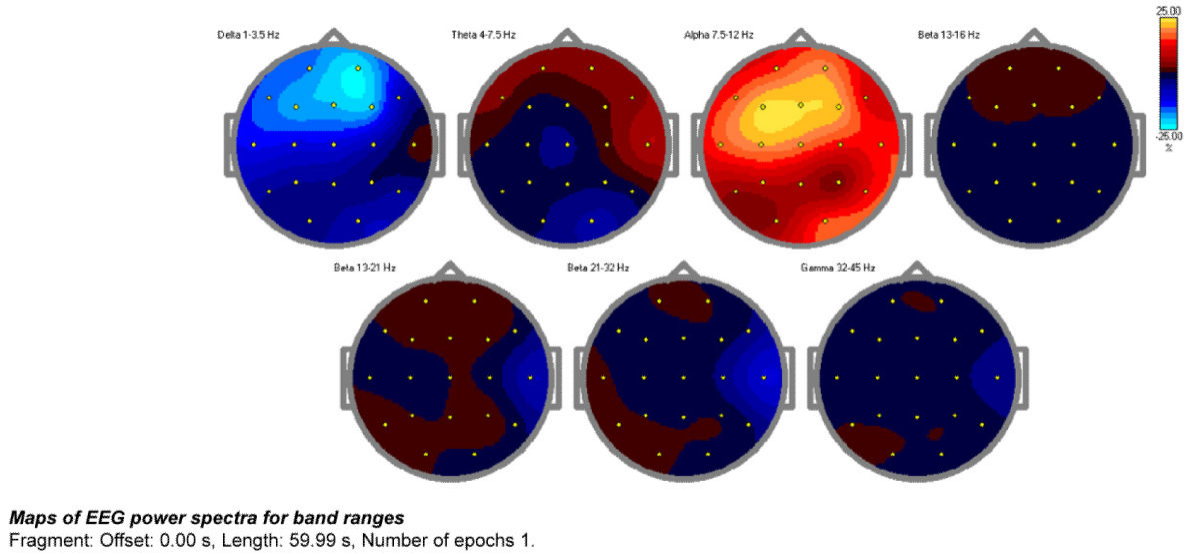
Validation experiment percentage change in microvolt’s squared Frequency band normalized power (% uV2) topographies for the EC conditions are presented below.

To validate these findings a percentage change in microvolts squared was performed as presented by the below data (Figure 3). The percentage of relative power change is congruent with the previous findings noting a 25% decrease in prefrontal delta and an increase in cortical potential marked by the prefrontal increase in activity throughout the theta and alpha spectrum. The overall alpha increase of 15-25% suggests an up regulation in post synaptic potentials noting that the brain has a more power to self-regulate.

## Discussion

In comparison of pre to post CES treatment the aforementioned absolute power ranges in these findings support the use of the CES technology in ameliorating the electrophysiological correlates of obsessive compulsion, anxiety, impulsivity, and cravings in addicted individuals [3-4,22-45].

The exact mechanism of NEAT-12 is unclear. However several preliminary studies have shown that CES alters various neurotransmitters or hormone levels in the brain [43]. One study demonstrated increased catecholamine levels in men and women and increased thyroxin production in men following log-term therapy with CES [36]. Others reported that platelet monoamine oxidase –B (MAO-B) activity and plasma concentration of Gamma –amino butyric acid (GABA) increased following CES, in conjunction with clinical improvement in anxiety and depression [31]. Shealy [44] has repeatedly shown that in both normal volunteers and in volunteers with treatment resistant depression, that CES was associated with significant elevations in cerebrospinal fluid and plasma serotonin respectively. CES has also been found to alter EEG readings in macaque monkeys during and after treatment. Alpha EEG waves were slowed following CES, which was associated with a reduction in adverse reactions to stressful stimuli [36,45]. In addition the observed attenuation of the high beta band with 100HZ CES treatment has clinical importance because this high beta band is associated with arousal, problem solving and stress [45]. Thus a slowing of alpha EEG waves coupled with a reduction of high beta bands together impact ones reaction to a stressful stimuli.

In our (ERB’s) clinical experience, long –term, daily 30-60 minutes CES treatment helped drug abusers to reduce, eliminate, and/or substitute the action of anti-depressants and benzodiazepines.

Based on the qEEG results, overall alpha increase of 15-25%, it is reasonable to expect that the use of this CES device by our abstinent alcoholic patient resulted in decreased stress. However, this pilot study must be confirmed by a large double-blind-sham controlled study in a well-characterized SUD population before anything definitive can be established.

To maintain homeostasis of the complex neuroanatomical brain system that utilizes neurotransmitters, poly-drug abusers who are known to be deficient in the neurotransmitters, may require continuous supplemental Neuroadaptogen Amino-Acid Therapy (NAAT) for neurotransmitter augmentation [46].

Our group Blum et al. [46]. have now completed a number of new studies using qEEG and functional magnetic resonance imaging (fMRI) showing that NAAT (specifically, Synaptose Complex Variant [KB220-Z]™) normalizes the electro-activity of the pre-frontal and cingulated gyrus region of the brain similar to the NEAT 12 devise in protracted poly-drug abusers with Reward Deficiency Syndrome (RDS) [47,48]. Use of this natural non-addicting, safe putative D2 agonist may find its place in recovery from (RDS) including addiction to psychoactive chemicals. We also cautiously suggest that long-term activation of dopaminergic receptors (i.e., DRD2 receptors) with NAAT will result in the proliferation of D2 receptors leading to enhanced “dopamine sensitivity” and an increased sense of happiness [49]. This is supported by numerous clinical trials on KB220 and awaits both fMRI and PET scanning to determine chronic effects of KB220-Z on numbers of D2 receptors and direct interaction at NAc.

Positive outcomes demonstrated by qEEG imaging in a randomized Triple-blind placebo controlled study involving oral KB220-Z, showed an increase of Alpha and low Beta activity [48]. Moreover, preliminary unpublished evidence derived from our fMRI studies in China indicate that an acute dose of KB220-Z compared to placebo in a double blinded study at rest activated the dopaminergic NAc as well as smoothing out abnormalities observed in the Putamen of heroin addicts. Additional addiction behavioral research involving cue induced reward site activation using fMRI is ongoing in China. These results will provide important information that could ultimately lead to significant improvement of recovery for victims of RDS having dopamine deficiency.

It is noteworthy, that alcohol may produce addiction by normalizing abnormal baseline states such as irritability, hyperexcitability, dysphoria, impulsiveness, or anxiety [50]. The results of animal studies have revealed that alcohol can induce sensitivity of neuronal membranes, chemically altered proteins, and adaptations to ion channels and factors related to the binding and release of neurotransmitters and neuromodulators including serotonin, pro-opiomelanocortins, enkephalins, gamma aminobutyric acid, glutamate receptors, and dopamine, norepinephrine and acetylcholine [51]. These effects could manifest via dopamine abducts with acetaldehyde leading to opioid – like actions and potential opioid receptor binding [52-54]. It is quite possible that modulation by NEAT-12 could offset impulsiveness, for example, as only one important target requiring intensive investigation.

While NEAT -12 are not strictly a CES device but a hybrid whereby we utilized current characteristics of TENS, we are encouraged that in larger cohort double-blinded studies it will produce similar CES effects as observed in this case study. A limitation to the use of this device is that its effects are only transient and must be coupled with natural Dopamine D2 agonistic therapy such as NAAT or newly developed variants for further longer term D2 activation in both the posterior VTA and Cingulate gyrus.

## Conclusion

The results of this pilot study are encouraging and we are proposing that NEAT-12 could provide adjunctive benefits to the addicted individual by reducing break-through aberrant cravings especially in conjunction with NAAT.

We propose following validation in large controlled studies, the systematic coupling of, RDS Inventory scale, genetic testing, personalized nutrigenomic (NAAT) solutions and NEAT 12, and Comprehensive Analysis of Reported Drugs™ (a medical monitoring system by Dominion Diagnostics) and miRNA monitoring (gene expression) as a potential RDS solution system if executed properly could revolutionize the RDS recovery field and ultimately provide an important contribution to the resolution of the ongoing difficulty we face in treating addiction and relapse [55,56].

## Figures and Tables

**Table 1:** QEEG, EEG changes in Substance Abuse.

Drug of Abuse	Measure	Outcome	Comment	Reference
**Alcohol**	qEEG and LORETA mapping	Increase in absolute and relative beta power and a decrease in alpha and delta/theta power.	Detoxified patients compared to normal controls	Saletu et al.[5]
**Alcohol**	EEG	Subjects with family history have reduced relative and absolute alpha power in occipital and frontal regions and increased relative beta in both regions.	Family history of alcoholism compared no family history	Finn and Justus [6]
**Alcohol**	EEG	Alcoholics differ in resting EEG coherence having lower frontal alpha and slow –beta coherence in males and females.	Heavy drinkers compared to light drinkers	Kaplan et al. [7]
**Alcohol**	EEG	In alcohol –dependent subjects found higher central alpha and slow –beta coherence, but lower parietal alpha and slow –beta coherence in males.	Alcohol -dependent compared to controls	Michael et al.[8]
**Alcohol**	EEG	Higher left-temporal alpha and slow beta coherence and higher slow-beta coherence at right –temporal and frontal electrode pairs in alcoholic males and females.	Alcohol -dependent compared to controls	Winterer et al. [9]
**Alcohol**	EEG	Moderate to heavy drinking is associated with differences in synchronization of brain activity during rest and mental rehearsal. Heavy drinkers displayed a loss of hemispheric asymmetry of EEG synchronization in the alpha and slow –beta band. Moderately and heavy drinking males also showed lower fast-beta band synchronization.	Comparison of moderate – heavy to Heavy drinking	De Bruin et al. [10]
**Marijuana**	EEG	Acute THC exposure produced transient increases in either posterior alpha power, decreases in mean alpha frequency or increase in alpha synchrony and decrease in relative power of beta.	Acute effects of THC	Struve et al. [11]
**Marijuana**	qEEG	Significant association between chronic marijuana use and topographic qEEG patterns of persistent “ alpha hyperfrontality” as well as reductions in alpha mean frequency. There was also elevated voltage of all non-alpha bands in chronic marijuana users. Finally there was a widespread decrease in the relative power of delta and beta activity over the frontal cortical regions in chronic marijuana users.	Chronic effects of THC exposure.	Struve et al. [12]
**Heroin**	qEEG	Qualitative changes were observed in more than 70% of heroin addicts in early abstinence and included low-voltage background activity with diminution of alpha rhythm , an increase in beta activity , and a large amount of low amplitude delta and theta waves in central regions. Also frequency shifts in fast alpha range at the frontal and central recording sites and a slowing of slow wave alpha mean frequency at the central, temporal, and occipital sites of recording heroin abusers who used heroin for at least 18 months.	Acute withdrawal	Polunia and Davydov et al. 13]
**Heroin**	qEEG	Abstinent alcoholics have an enhanced fast beta power compared to healthy controls	Alcoholics compared to healthy controls	Franken et al. [14]
**Heroin**	EEG	Elevated synchrony within beta frequency during short term heroin withdrawal may reflect a state of CNS activation toward reward –seeking behavior, with this being a prerequisite to relapse among opiate drug dependent patients.	Polydrug abusers with emphasis on heroin abuse.	Bauer et al. [15]
**Cocaine**	EEG	Acute effects of cocaine include increase in beta activity, increase in delta, increase in frontal alpha as well as an increase in alpha wave EEG associated with bursts of cocaine –induce euphoria.	Human studies	Prichep et al. [16] Alper[17]
**Cocaine**	qEEG	During protracted abstinence from cocaine qEEG effects include long-lasted increases in alpha and beta bands together with reduced activity in delta and theta bands.	Several studies reported similar effects on withdrawal.	Roemer et al. [18]
**Cocaine**	qEEG	Cocaine produced a rapid increase in absolute theta, alpha and beta power over the prefrontal cortex, up to 25 minutes after drug administration. The increase in theta power was correlated with a positive drug high, and the increase in alpha power was correlated with anxiety. Also an increase in delta coherence over the prefrontal cortex correlated with nervous energy.	qEEG profiles in cocaine- dependent patients in response to an acute, single- blind, self –administered dose of smoked cocaine base (50 mg) versus placebo.	Reid et al. [19]
**Cocaine**	qEEG	Changes occur 5-14 days after last reported crack cocaine use induced changes in brain function. These changes lasted up to six months.	Subjects with cocaine dependence have persistent changes in brain function	Venneman et al. [20]
**Cocaine**	qEEG	qEEG techniques demonstrate an association between beta activity in the spontaneous EEG and relapse in cocaine abuse.	qEEG changes associate with relapse	Ceballos et al. [21]

Source: Miller et al. Post Graduate Medicine (in press) with permission.

**Table 2:** NEAT-12 programs.

Program	Name	MicroAmperage	Frequency	*Pulse Width	Comment
1	Low Freq Steady	25	0.44 Hz	1.12s	This program is for those individuals that have a simple diagnosis of stress, anxiety or insomnia in aftercare treatment.
2	Med Freq Steady	25	2.5 Hz	.02s	This program is for those individuals that have a moderate diagnosis of stress, anxiety or insomnia in aftercare treatment.
3	High Freq Steady	25	7 Hz	0.714s	This program is for those individuals that have a severe diagnosis of stress, anxiety or insomnia in aftercare treatment.
4	Low Freq modulated	25	0.44 Hz	High 1.85s Low .33s	This program is for those individuals that have a simple diagnosis of stress, anxiety or drug addition in aftercare treatment.
5	Med Freq modulated	25	2.5 Hz	High .32s Low .06s	This program is for those individuals that have a moderate diagnosis of stress, anxiety or drug addition in aftercare treatment.
6	High Freq modulated	25	7 Hz	High .114s Low .021s	This program is for those individuals that have a severe diagnosis of stress, anxiety or drug addition in aftercare treatment.
7	Low Freq Steady	100	0.44 Hz	1.12s	This program is for those individuals that have a simple diagnosis of stress, anxiety or insomnia that are in acute treatment.
8	Med Freq Steady	100	2.5 Hz	.02s	This program is for those individuals that have a moderate diagnosis of stress, anxiety or insomnia that are in acute treatment.
9	High Freq Steady	100	7 Hz	0.714s	This program is for those individuals that have a severe diagnosis of stress, anxiety or insomnia that are in acute treatment.
10	Low Freq modulated	100	0.44 Hz	High 1.85s Low .33s	This program is for those individuals that have a simple diagnosis of stress, anxiety or drug addition that are in acute treatment.
11	Med Freq modulated	100	2.5 Hz	High .32s Low .06s	This program is for those individuals that have a moderate diagnosis of stress, anxiety or drug addition that are in acute treatment.
12	High Freq modulated	100	7 Hz	High .114s Low .021s	This program is for those individuals that have a severe diagnosis of stress, anxiety or drug addition that are in acute treatment.

* The measurement of the pulse width reported is without a load. Also, in a non-modulated program the pulse width measurement of individual pulses, the pulse duration range is 1 microsecond to 1000 microseconds with a 1000 ohm load.

## References

[1] Gilula MF (2007). Cranial electrotherapy stimulation and fibromyalgia. Expert Rev Med Devices.

[2] Zaghi S, Acar M, Hultgren B, Boggio PS, Fregni F (2010). Noninvasive brain stimulation with low-intensity electrical currents: putative mechanisms of action for direct and alternating current stimulation. Neuroscientist.

[3] Electrosleep and cerebral electrotherapy. Med Lett Drugs Ther.

[4] Post RM, Trimble MR, Pippenger CE (1989). Clinical use of anti-convulsive in psychiatric disorders.

[5] Saletu B, Anderer P, Saletu-Zyhlarz GM, Arnold O, Pascual-Marqui RD (2002). Classification and evaluation of the pharmacodynamics of psychotropic drugs by single-lead pharmaco-EEG, EEG mapping and tomography (LORETA). Methods Find Exp Clin Pharmacol.

[6] Finn PR, Justus A (1999). Reduced EEG alpha power in the male and female offspring of alcoholics. Alcohol Clin Exp Res.

[7] Kaplan RF, Glueck BC, Hesselbrock MN, Reed HB (1985). Power and coherence analysis of the EEG in hospitalized alcoholics and nonalcoholic controls. J Stud Alcohol.

[8] Michael A, Mirza KA, Mukundan CR, Channabasavanna SM (1993). Interhemispheric electroencephalographic coherence as a biological marker in alcoholism. Acta Psychiatr Scand.

[9] Winterer G, Enoch MA, White KV, Saylan M, Coppola R (2003). EEG phenotype in alcoholism: increased coherence in the depressive subtype. Acta Psychiatr Scand.

[10] de Bruin EA, Stam CJ, Bijl S, Verbaten MN, Kenemans JL (2006). Moderate-to-heavy alcohol intake is associated with differences in synchronization of brain activity during rest and mental rehearsal. Int J Psychophysiol.

[11] Struve FA, Straumanis JJ, Patrick G, Price L (1989). Topographic mapping of quantitative EEG variables in chronic heavy marihuana users: empirical findings with psychiatric patients. Clin Electroencephalogr.

[12] Struve FA, Manno BR, Kemp P, Patrick G, Manno JE (2003). Acute marihuana (THC) exposure produces a “transient” topographic quantitative EEG profile identical to the “persistent” profile seen in chronic heavy users. Clin Electroencephalogr.

[13] Polunina AG, Davydov DM (2004). EEG spectral power and mean frequencies in early heroin abstinence. Prog Neuropsychopharmacol Biol Psychiatry.

[14] Franken IH, Stam CJ, Hendriks VM, van den Brink W (2004). Electroencephalographic power and coherence analyses suggest altered brain function in abstinent male heroin-dependent patients. Neuropsychobio.

[15] Bauer LO (2001). Predicting relapse to alcohol and drug abuse via quantitative electroencephalography. Neuropsychopharmacol.

[16] Prichep LS, Alper KR, Sverdlov L, Kowalik SC, John ER (2002). Outcome related electrophysiological subtypes of cocaine dependence. Clin Electroencephalogr.

[17] Alper KR (1999). The EEG and cocaine sensitization: a hypothesis. J Neuropsychiatry Clin Neurosci.

[18] Roemer RA, Cornwell A, Dewart D, Jackson P, Ercegovac DV (1995). Quantitative electroencephalographic analyses in cocaine-preferring polysubstance abusers during abstinence. Psychiatry Res.

[19] Reid MS, Flammino F, Howard B, Nilsen D, Prichep LS (2006). Topographic imaging of quantitative EEG in response to smoked cocaine self-administration in humans. Neuropsychopharmacology.

[20] Venneman S, Leuchter A, Bartzokis G, Beckson M, Simon SL (2006). Variation in neurophysiological function and evidence of quantitative electroencephalogram discordance: predicting cocaine-dependent treatment attrition. J Neuropsychiatry Clin Neurosci.

[21] Ceballos NA, Bauer LO, Houston RJ (2009). Recent EEG and ERP findings in substance abusers. Clin EEG Neurosci.

[22] Kirsch DL, Smith RB (2000). The use of cranial electrotherapy stimulation in the management of chronic pain: A review. Neuro Rehabilitation.

[23] Solomon S, Elkind A, Freitag F, Gallagher RM, Moore K (1989). Safety and effectiveness of cranial electrotherapy in the treatment of tension headache. Headache.

[24] Smith RB, Tiberi A, Marshall J (1994). The use of cranial electrotherapy stimulation in the treatment of closed-head-injured patients. Brain Inj.

[25] Long RC (1966). Electrosleep therapy. Some results with the use of electrically induced sleep in the treatment of psychiatric patients. J Kans Med Soc.

[26] Buckman C, Pinsley I, Fenichel M (1957). Electrosleep therapy in psychosis. Dis Nerv Syst.

[27] Bystritsky A, Kerwin L, Feusner J (2008). A pilot study of cranial electrotherapy stimulation for generalized anxiety disorder. J Clin Psychiatry.

[28] Flemenbaum A (1974). Cerebral electrotherapy (electrosleep): an open-clinical study with a six month follow-up. Psychosomatics.

[29] Moore JA, Mellor OS, Standage KF, Strong H (1975). A double-blind study of electrosleep for anxiety and insomnia. Biol Psychiatry.

[30] Philip P, Demotes-Mainard J, Bourgeois M, Vincent JD (1991). Efficiency of transcranial electrostimulation on anxiety and insomnia symptoms during a washout period in depressed patients. A double-blind study. Biol Psychiatry.

[31] Klawansky S, Yeung A, Berkey C, Shah N, Phan H (1995). Meta-analysis of randomized controlled trials of cranial electrostimulation. Efficacy in treating selected psychological and physiological conditions. J Nerv Ment Dis.

[32] Braverman ER, Smith R, Smayda R, Blum K (1990). Modification of P330 amplitude and other electrophysiological parameters of drug abuse by cranial electrical stimulation. Cur Therap Res.

[33] Schmitt R, Capo T, Frazier H, Boren D (1984). Cranial electrotherapy stimulation treatment of cognitive brain dysfunction in chemical dependence. J Clin Psychiatry.

[34] Schmitt R, Capo T, Boyd E (1986). Cranial electrotherapy stimulation as a treatment for anxiety in chemically dependent persons. Alcohol Clin Exp Res.

[35] Krupitsky EM, Burakov AM, Karandashova GF, Katsnelson JaS, Lebedev VP (1991). The administration of transcranial electric treatment for affective disturbances therapy in alcoholic patients. Drug Alcohol Depend.

[36] Jarzembski WB (1985). Electrical stimulation and substance abuse treatment. Neurobehav Toxicol Teratol.

[37] Demotes-Mainard J, Philip P, Jalfre M, Vincent JD (1990). Transcerebral electrostimulation in hypnotic drug withdrawal. Encephale.

[38] Pickworth WB, Fant RV, Butschky MF, Goffman AL, Henningfield JE (1997). Evaluation of cranial electrostimulation therapy on short-term smoking cessation. Biol Psychiatry.

[39] Auriacombe M, Tignol J, Le Moal M, Stinus L (1990). Transcutaneous electrical stimulation with Limoge current potentiates morphine analgesia and attenuates opiate abstinence syndrome. Biol Psychiatry.

[40] Stinus L, Auriacombe M, Tignol J, Limoge A, Le Moal M (1990). Transcranial electrical stimulation with high frequency intermittent current (Limoge’s) potentiates opiate-induced analgesia: blind studies. Pain.

[41] Alling FA, Johnson BD, Elmoghazy E (1990). Cranial electrostimulation (CES) use in the detoxification of opiate-dependent patients. J Subst Abuse Treat.

[42] Braverman ER, Blum K, Smayda RJ (1990). A commentary on brain mapping in 60 substance abusers: Can the potential for drug abuse be predicted and prevented by treatment?. Curr Ther Res.

[43] Ferdjallah M, Bostick FX, Barr RE (1996). Potential and current density distributions of cranial electrotherapy stimulation (CES) in a four-concentric-spheres model. IEEE Trans Biomed Eng.

[44] Shealy CN (2003). Transcutaneous electrical nerve stimulation: the treatment of choice for pain and depression. J Altern Complement Med.

[45] Schroeder MJ, Barr RE (2001). Quantitative analysis of the electroencephalogram during cranial electrotherapy stimulation. Clin Neurophysiol.

[46] Blum K, Oscar-Berman M, Stuller E, Miller D, Giordano J (2012). Neurogenetics and Nutrigenomics of Neuro-Nutrient Therapy for Reward Deficiency Syndrome (RDS): Clinical Ramifications as a Function of Molecular Neurobiological Mechanisms. J Addict Res Ther.

[47] Miller D, Manka M, Miller M, Stokes S, Allen C (2010). Acute Intravenous Synaptamine Complex [KB220]™ Variant “Normalizes” Abnormal Neurological Activity in Protracted Abstinence of Alcohol and Opiate Patients Using Quantitative Electroencephalographic (QEEG) and Neurotransmitter Genetic Analysis: Pilot Two Case Reports. Postgraduate Medicine.

[48] Blum K, Chen TJH, Morse S, Giordano J, Chen ALC (2010). Overcoming qEEG abnormalities and reward gene deficits during protracted abstinence in psychostimulant abusers utilizing putative dopamine D2 agonist therapy. Part 2. Postgraduate Medicine.

[49] Blum K, Chen AL, Chen TJ, Braverman ER, Reinking J (2008). Activation instead of blocking mesolimbic dopaminergic reward circuitry is a preferred modality in the long term treatment of reward deficiency syndrome (RDS): a commentary. Theor Biol Med Model.

[50] Archer T, Oscar-Berman M, Blum K, Gold M (2012). Neurogenetics and Epigenetics in Impulsive Behaviour: Impact on Reward Circuitry. J Genet Syndr Gene Ther.

[51] Shan HQ, Hammarback JA, Godwin DW (2013). Ethanol inhibition of a T-type Ca^2^+ channel through activity of protein kinase C. Alcohol Clin Exp Res.

[52] Blum K, Hamilton MG, Hirst M, Wallace JE (1978). Putative role of isoquinoline alkaloids in alcoholism: a link to opiates. Alcohol Clin Exp Res.

[53] Blum K, Wallace JE, Ryback RS, Geller I (1972). Diethanolamine: a possible weak agonist-antagonist to ethanol. Eur J Pharmacol.

[54] Blum K, Sheridan PJ, Wood RC, Braverman ER, Chen TJ (1995). Dopamine D2 receptor gene variants: association and linkage studies in impulsive-addictive-compulsive behaviour. Pharmacogenetics.

[55] Blum K, Chen TJ, Downs BW, Bowirrat A, Waite RL (2009). Neurogenetics of dopaminergic receptor supersensitivity in activation of brain reward circuitry and relapse: proposing “deprivation- amplification relapse therapy” (DART). Postgrad Med.

[56] Dahlgren A, Wargelius HL, Berglund KJ, Fahlke C, Blennow K (2011). Do alcohol-dependent individuals with DRD2 A1 allele have an increased risk of relapse? A pilot study. Alcohol Alcohol.

